# Extramedullary hematopoiesis contributes to enhanced erythropoiesis during pregnancy via TGF-β signaling

**DOI:** 10.3389/fimmu.2023.1295717

**Published:** 2023-11-17

**Authors:** Yao Fu, Zhengjuan Li, Wen Lin, Jingxin Yao, Xiang Jiang, Qun Shu, Xiaoyuan Mao, Jiaoqin Tu, Xinyuan Liang, Liping Li

**Affiliations:** ^1^ Department of Obstetrics, Shenzhen People’s Hospital, The Second Clinical Medical College, Jinan University, Shenzhen, China; ^2^ Post-doctoral Scientific Research Station of Clinical Medicine, Jinan University, Guangzhou, China; ^3^ Department of Obstetrics, Shanghai First Maternity and Infant Hospital, Tongji University School of Medicine, Shanghai, China; ^4^ South China University of Technology School of Medicine, Guangzhou, China; ^5^ Guangzhou Women and Children’s Medical Center, Guangzhou Medical University, Guangzhou, China; ^6^ Department of Obstetrics and Gynecology, The Eighth Affiliated Hospital, Sun Yat-sen University, Shenzhen, China

**Keywords:** CD71^+^ erythroid cells (CECs), erythropoiesis, extramedullary hematopoiesis, transforming growth factor-β, splenic stromal cells, pregnancy

## Abstract

Red blood cells are the predominant cellular component in human body, and their numbers increase significantly during pregnancy due to heightened erythropoiesis. CD71^+^ erythroid cells (CECs) are immature red blood cells, encompassing erythroblasts and reticulocytes, constitute a rare cell population primarily found in the bone marrow, although they are physiologically enriched in the neonatal mouse spleen and human cord blood. Presently, the mechanisms underlying the CECs expansion during pregnancy remain largely unexplored. Additionally, the mechanisms and roles associated with extramedullary hematopoiesis (EMH) of erythroid cells during pregnancy have yet to be fully elucidated. In this study, our objective was to examine the underlying mechanisms of erythroid-biased hematopoiesis during pregnancy. Our findings revealed heightened erythropoiesis and elevated CECs in both human and mouse pregnancies. The increased presence of transforming growth factor (TGF)-β during pregnancy facilitated the differentiation of CD34^+^ hematopoietic stem and progenitor cells (HSPCs) into CECs, without impacting HSPCs proliferation, ultimately leading to enhanced erythropoiesis. The observed increase in CECs during pregnancy was primarily attributed to EMH occurring in the spleen. During mouse pregnancy, splenic stromal cells were found to have a significant impact on splenic erythropoiesis through the activation of TGF-β signaling. Conversely, splenic macrophages were observed to contribute to extramedullary erythropoiesis in a TGF-β-independent manner. Our results suggest that splenic stromal cells play a crucial role in promoting extramedullary erythropoiesis and the production of CECs during pregnancy, primarily through TGF-β-dependent mechanisms.

## Introduction

Hematopoietic stem cells (HSCs) possess the distinctive ability to undergo self-renewal and differentiate into all blood cell lineages within the hematopoietic system (1, 2). Erythropoiesis, the process by which the human body generates approximately 2 × 10^11^ red blood cells per day, is a tightly regulated phenomenon (3). Throughout erythropoiesis, HSCs undergo differentiation into megakaryocyte-erythrocyte progenitors (MEPs), which subsequently differentiate into burst-forming unit-erythroid (BFU-E) progenitors and colony-forming unit erythroid (CFU-E) progenitors (4). At the late stage of erythropoiesis, CFU-E progenitor cells differentiate into proerythrocytes and then into basophilic erythrocytes, polychromatic erythrocytes, positive erythrocytes, reticulocytes and mature erythrocytes in turn (5).

Steady-state erythropoiesis primarily takes place in the bone marrow and is governed by the actions of erythropoietin (EPO), stem cell factor (SCF), and interleukin (IL)-3 (6). However, during periods of physiological stress, erythropoiesis in humans occurs predominantly in the bone marrow, with additional contributions from the spleen. In contrast, stress-induced erythropoiesis in mice primarily occurs in the spleen (6). The process of actively differentiating hematopoietic cells outside the bone marrow is extramedullary hematopoiesis (EMH), which is believed to significantly contribute to hematopoiesis under stress or disease situations (7, 8). The spleen is an important organ for EMH under stress states (9). The extensive proliferation of erythroid progenitor cells plays a crucial role in this response, which is closely associated with the splenic microenvironment (9).

CD71^+^ erythroid cells (CECs) are immature red blood cells, which consist of erythroblasts and reticulocytes (10) and express transferrin receptor I (CD71) and glycoprotein A (CD235a) in humans, or CD71 and glycoprotein A-related protein (Ter119) in mice (11). While CECs are typically scarce in healthy adults and primarily found in the bone marrow, they are naturally enriched in the spleen of neonatal mice and in human cord blood (11). Their numbers also increase significantly under certain stress conditions, including tumor and anemia (12). In a mouse tumor-bearing model, hemoglobin concentration and hematocrit gradually decrease as the tumor progresses, and the hypoxic environment and tumor-induced anemia increase blood EPO concentration (13), accompanied by the aggregation of CECs in the spleen and liver other than in the bone marrow (12–14).

Red blood cells are the most abundant cellular component in the human body, and their number reaches 25 × 10^12^, accounting for more than 80% of the total number of human cells (15). In addition to transporting oxygen, another important function of erythroid cells is to regulate immune responses (16). The role of CECs in neonatal infection has been extensively studied by the Elahi group (10, 17, 18). As discovered by Elahi et al. in 2013 (10), CECs have been found to be abundant in neonatal mice and human cord blood and possess unique immunosuppressive properties. Subsequently, they found that CECs in neonatal mouse spleen inhibit the immune response against *pertussis* infection, and CD71^+^CD235a^+^ cells in human cord blood inhibit T and B cell function *in vitro* (17, 18). Additionally, CECs derived from cancer patients have been shown to hinder the proliferation and differentiation of CD4^+^ T cells, as well as the proliferation and cytotoxicity of CD8^+^ T cells (14).

The establishment of maternal-fetal tolerance and pregnancy maintenance necessitates intricate regulation of the immune system (19). CECs in the field of reproduction have not been studied until recently. It has been reported that women and female mice are enriched with CECs (20), and increased estrogen and 27-hydroxycholesterol in pregnant mice act on estrogen receptor α and promote the division of HSCs and the expansion of CECs in the spleen (21, 22). During pregnancy, the expansion of CECs is observed in the peripheral blood and placenta of humans, as well as in the placenta and spleen of mice (23). Furthermore, it has been demonstrated that CECs present in the peripheral blood of pregnant women inhibit the proliferation of CD4^+^ and CD8^+^ T cells *in vitro*.

During pregnancy, erythropoiesis becomes active, and erythrocytes significantly expand (24). In addition, recent findings highlight the significance of CECs expansion and the establishment of maternal-fetal immunity in pregnancy, shedding new light on the role of CECs as a crucial cell population involved in creating and maintaining maternal-fetal tolerance. To date, the mechanisms underlying the CECs expansion during pregnancy remain largely unexplored. Additionally, the mechanisms and roles associated with extramedullary erythropoiesis during pregnancy have yet to be fully elucidated. Transform growth factor (TGF)-β is one of the most important cytokines in maintaining maternal-fetal tolerance during pregnancy (25), and it has been found to play an important role in regulating HSCs erythroid lineage differentiation (26, 27).

The current study provides evidence of a notable increase in CECs during both human and mouse pregnancies. In mouse pregnancy, the spleen plays a crucial role in generating CECs through extramedullary erythropoiesis, as opposed to the bone marrow. Furthermore, our findings indicate that splenic stromal cells play a significant role in facilitating the differentiation of murine HSCs into CECs, primarily via the TGF-β/Smad pathway. These results suggest that the splenic niche plays a vital role in promoting extramedullary erythropoiesis and CECs production during pregnancy, with TGF-β serving as a key mediator.

## Materials and methods

### Human subjects

Peripheral blood from pregnant women (n = 30) and non-pregnant healthy women (n = 30) in Shenzhen People’s Hospital were obtained. The umbilical cord blood samples were obtained from healthy full-term deliveries. The samples were processed within three hours of collection. The human studies conducted in this research were subjected to review and approval by the Institutional Review Board of Shenzhen People’s Hospital. Prior to their participation, all study participants provided written informed consent.

### Animals

C57BL/6 mice were bred in a pathogen-free environment at the Laboratory Animal Center of Shenzhen People’s Hospital. Each female mouse, aged between 10-12 weeks, was co-caged with a male mouse. The detection of a vaginal plug marked the initiation of embryonic day 0.5 (E0.5). All animal experiments conducted in this study were granted approval by the Animal Care and Use Committee of Jinan University.

### Isolation and expansion of human CD34+ hematopoietic stem and progenitor cells (HSPCs)

Peripheral blood mononuclear cells (PBMCs) and umbilical cord blood mononuclear cells (UBMCs) were initially separated through centrifugation on a Ficoll-Paque Premium (1.077 g/mL density gradient medium; Cytiva, Piscataway, NJ, USA). Subsequently, CD34^+^ cells were purified using a direct CD34 progenitor cell isolation kit as per the manufacturer’s guidelines (Miltenyi Biotec, Bergisch Gladbach, Germany). In some experiments, PBMCs were stained to analyze HSPCs subsets using flow cytometry.

### Erythroid differentiation from human CD34^+^ HSPCs *in vitro*

Fresh isolated human UBMC-derived CD34^+^ cells were induced to differentiate into erythroid cells *in vitro*. Briefly, cells (1 × 10^4^ cells/mL) were added to a 24-well plate and cultured in a serum-free erythroid differentiation medium, consisting of the StemSpan SFEM II medium (Stem Cell Technologies, Vancouver, Canada), 50 ng/mL SCF (PeproTech, Rocky Hill, NJ, USA), 5 ng/mL IL-3 (BioLegend, San Diego, CA, USA), and 6 U/mL EPO (PeproTech). Different concentrations (0.01, 0.1, 1, and 10 ng/mL) of recombinant human TGF-β (rhTGF-β; R&D Systems, Minneapolis, MN, USA) were added to the medium. Medium was half-changed every two days. On day 7, cells were reseeded at a density of 5 × 10^4^ cells/mL. The cells were harvested on day 7 or day 11 and analyzed for the expression of CD71 and CD235a using flow cytometry or real-time polymerase chain reaction (RT-PCR). In selected experiments, cells were cultured in the presence of estradiol (0.1, 1, and 10 ng/ml; MedChemExpress, Princeton, NJ, USA), progesterone (10, 100, and 1000 ng/ml; MedChemExpress), or human chorionic gonadotropin β (β-hCG, 1, 10, and 100 IU/mL; R&D Systems). Each set of experiments was replicated three times.

### Isolation and expansion of mouse Lineage^-^Sca-1^+^c-Kit^+^ (LSK) cells

Mouse LSK hematopoietic stem cells were isolated from mouse bone marrow. Briefly, mouse bone marrow mononuclear cells were first isolated. Lin^-^ cells were purified using a Direct Lineage Depletion kit (Miltenyi Biotec). Lin^-^ cells were then incubated with anti-c-Kit microbeads (Miltenyi Biotec), and Lin^-^c-Kit^+^ cells were sorted using the miniMACS (Miltenyi Biotec) separator. Lin^-^c-Kit^+^ cells were then stained with fluorescence-conjugated anti-Sca-1 Ab, and Lin^-^Sca-1^+^c-Kit^+^ LSK cells were sorted using the Sony LE-SH800ZBP flow cytometer (Tokyo, Japan). Sorted cells were analyzed using flow cytometry, and the cell purity was > 99%. For *in vitro* expansion, LSK cells were plated at a concentration of 2 × 10^4^ cells/mL in a 24-well plate with StemSpan SFEM supplemented with 10% fetal bovine serum (FBS; Hyclone, Piscataway, NJ, USA), 100 ng/mL SCF, 100 ng/mL Fms-like tyrosine kinase 3 (Flt-3; BioLegend, San Diego, CA, USA), and 100 ng/mL thrombopoietin (TPO, PeproTech). Cells were cultured at 37°C in 5% CO_2_ for 3 days.

### Isolation of mouse splenic stromal cells

Spleens of non-pregnant mice or pregnant mice at E12.5 were harvested, cut into small pieces, and then filtered through a 40 µm strainer (Thermo Fisher Scientific, Waltham, MA, USA). Connective tissues were collected and digested with a digesting medium containing 1 mg/mL collagenase IV (Sigma-Aldrich, St. Louis, MO, USA), 500 µg/mL collagenase D (Roche, Basel, Switzerland), 40 µg/mL DNase I (Roche) and 2% FBS for about 20 min at 37°C in a shaking water bath. Digested cells were washed with PBS and filtered through the 40 µm cell strainer. The cells were resuspended and cultured at 37°C in 5% CO_2_ in a T25 flask (Thermo Fisher Scientific) containing dulbecco’s modified eagle medium (DMEM; Hyclone) supplemented with 10% FBS, 100 U/mL penicillin (Hyclone) and 100 μg/mL streptomycin (Hyclone) until about 90% confluence. Cells were then collected and incubated with anti-CD45 microbeads (Miltenyi Biotec). CD45^-^ stromal cells were purified using the miniMACS separator.

### *In vitro* co-cultures

Bone marrow LSK cells (1 × 10^4^ cells/mL) were co-cultured with CD45^-^ splenic stromal cells (1 × 10^4^ cells/mL) or macrophages (2 × 10^4^ cells/mL) in an erythroid differentiation system for 11 days. Medium was half-changed every two days. In some experiments, TGF-β pathway inhibitors, including 1 μM SB431542 (a TGF-β receptor inhibitor; MedChemExpress), 1 μM Galunisertib (a TGF-β receptor inhibitor; MedChemExpress), 1 μM ITD-1 (a Smad2/3 inhibitor; MedChemExpress), and 10 μg/mL anti-mouse TGF-β Ab (Bioxcell, Lebanon, NH, USA), were added to the co-culture systems. The frequencies of CECs in the co-cultures were monitored using flow cytometry.

### Flow cytometry

For cell-surface staining, single-cell suspensions in PBS with 1% FBS were stained with fluorescence-conjugated Abs for 20 min on ice. To analyze intracellular factors, cells were incubated with fluorescence-conjugated Abs for 20 min at 4°C. The cells were washed, fixed, and resuspended in permeabilization wash buffer, followed by incubation with fluorescence-conjugated antibodies targeting the desired factors. Data were acquired on a Cytek spectrum flow cytometer (Cytek Bioscience, Fremont, CA, USA) and analyzed using FlowJo software (Version 10.5.3; Ashland, OR, USA). Fluorescence-conjugated Abs, fixation buffer, and permeabilization wash buffer were all purchased from BioLegend. The fluorescence-conjugated Abs used in this study are summarized in **Supplementary Table 1**.

### Enzyme-linked immunosorbent assay (ELISA)

The concentrations of extracellular TGF-β in the peripheral blood from pregnant and non-pregnant women or mice were measured using ELISA according to the manufacturer’s instructions (ExCell Bio, Shanghai, China). Three to five samples were collected within each experimental group, and each experiment was repeated three times.

### Western blot

Tissues or cells were mashed and lysed in lysis buffer (Beyotime Institute of Biotechnology, Shanghai, China) for 10 minutes on ice. The resulting mixture was centrifuged at 13,000 *g* for 10 minutes at 4°C, and the lysates were collected. The protein concentrations were determined using the Pierce^®^ BCA protein assay kit (Thermo Fisher Scientific). Approximately 30 μg of protein was separated on a 4%-12% Bis-Tris Gel (Genscript, Shanghai, China) and subsequently transferred onto a PVDF membrane (Millipore, Billerica, MA, USA). The membrane was blocked and incubated with a rabbit-anti-mouse Ab against TGF-β (1: 1000; Abcam, Cambridge, UK) overnight at 4°C. The membrane was then washed and incubated with horseradish peroxidase-coupled goat-anti-rabbit IgG (1: 5000; Thermo Fisher Scientific). Afterward, immunoreactive bands were developed using the SuperSignal™ Chemiluminescent Substrate kit (Thermo Fisher Scientific). The expression of β-actin (1: 1000; Cell Signaling Technology, Danvers, MA, USA) was used as the internal control. Bands were quantified using the ImageJ 1.52H software (National Institutes of Health, USA).

### Construction of a mouse model of splenectomy

The pregnant mouse at E9.5 was anesthetized with isoflurane (Sigma-Aldrich), and the abdominal cavity of the mouse was surgically accessed, followed by ligation of the splenic vessels and careful removal of the spleen. In the sham surgery group, the abdomen was opened and the para-splenic fat was ligated and removed, while the spleen remained intact. Four to five mice were used within each experimental group. After three days, the mouse was euthanized and the peripheral blood, bone marrow, and decidua were collected for subsequent flow cytometric analysis.

### Statistical analysis

All statistical analyses were conducted using SPSS 19.0 software (Chicago, IL, USA). Data were assessed using one-way ANOVA with Bonferroni test for comparisons among three or more groups, or independent Student’s *t*-test and paired *t*-test for comparisons between two groups. The results are given as the mean ± SD. A *P* value of < 0.05 was considered statistically significant among the analyzed groups.

Other detailed information is provided in the **Supplemental Information file**.

## Results

### Comparison of circulating HPSCs subsets in non-pregnant and pregnant women

**Figure 1:**
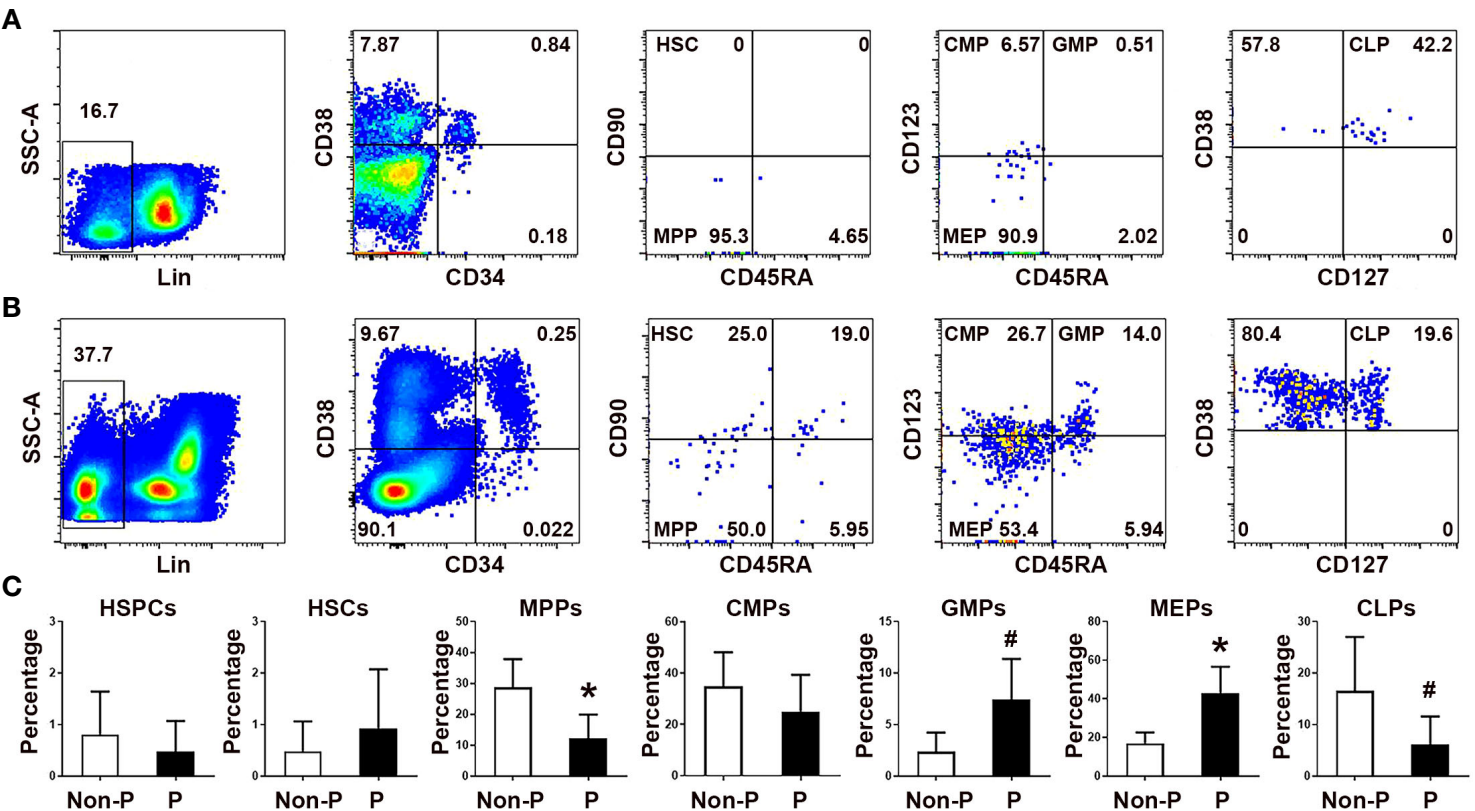
Comparison of the frequencies of circulating HSPC subsets in non-pregnant and pregnant women. Representative flow cytometric scatter plots depicting Lin^-^CD34^+^ HSPCs and their respective subgroups in non-pregnant **(A)** and pregnant women **(B)** were presented. **(C)** A graphical summary illustrating the percentages of HSPCs and their subgroups were shown. Statistical analysis was performed using independent Student’s *t*-test. The results were expressed as mean ± SD. ^#^ *P* < 0.05 and ^*^ *P* < 0.01 vs. the non-pregnant women. Non-P, non-pregnant women; P, pregnant women.

We first evaluated the frequencies of peripheral blood HSPCs subsets in non-pregnant and pregnant women. As shown in **Figure 1**, the frequencies of peripheral blood Lin^-^CD34^+^ HSPCs to Lin^-^ cells, Lin^-^CD34^+^CD38^+^ HSCs to HSPCs, and common myeloid progenitors (CMPs) to HSPCs did not differ significantly between non-pregnant and pregnant women. Compared with non-pregnant women, although the frequency of blood multipotent progenitors (MPPs) significantly decreased (*P* < 0.01), the frequencies of granulocyte-monocyte progenitors (GMPs; *P* < 0.05) and megakaryocyte-erythrocyte progenitors (MEPs; *P* < 0.01) significantly increased. In contrast, we observed a marked decrease of common lymphoid progenitors (CLPs; *P* < 0.01) in pregnant women compared to non-pregnant women.

We also compared the numbers of peripheral blood HSPCs subsets between non-pregnant and pregnant women (**Supplementary Figure 1**). Although the frequencies of HSPCs did not differ between non-pregnant and pregnant women, the number of HSPCs in the peripheral blood of pregnant women significantly increased compared with non-pregnant donors (*P* < 0.01). In accordance with the cell frequency results, the numbers of GMPs (*P* < 0.05) and MEPs (*P* < 0.01) in pregnant women were significantly increased compared with those in non-pregnant women. In addition, the numbers of CMPs (*P* < 0.01) and CLPs (*P* < 0.05) were also significantly increased in pregnant women compared with controls. Our results suggest that hematopoiesis, including myelopoiesis and erythropoiesis, is enhanced in pregnant women, and erythropoiesis is the most active in pregnancy.

### Increased frequencies of peripheral blood CECs during human pregnancy

**Figure 2:**
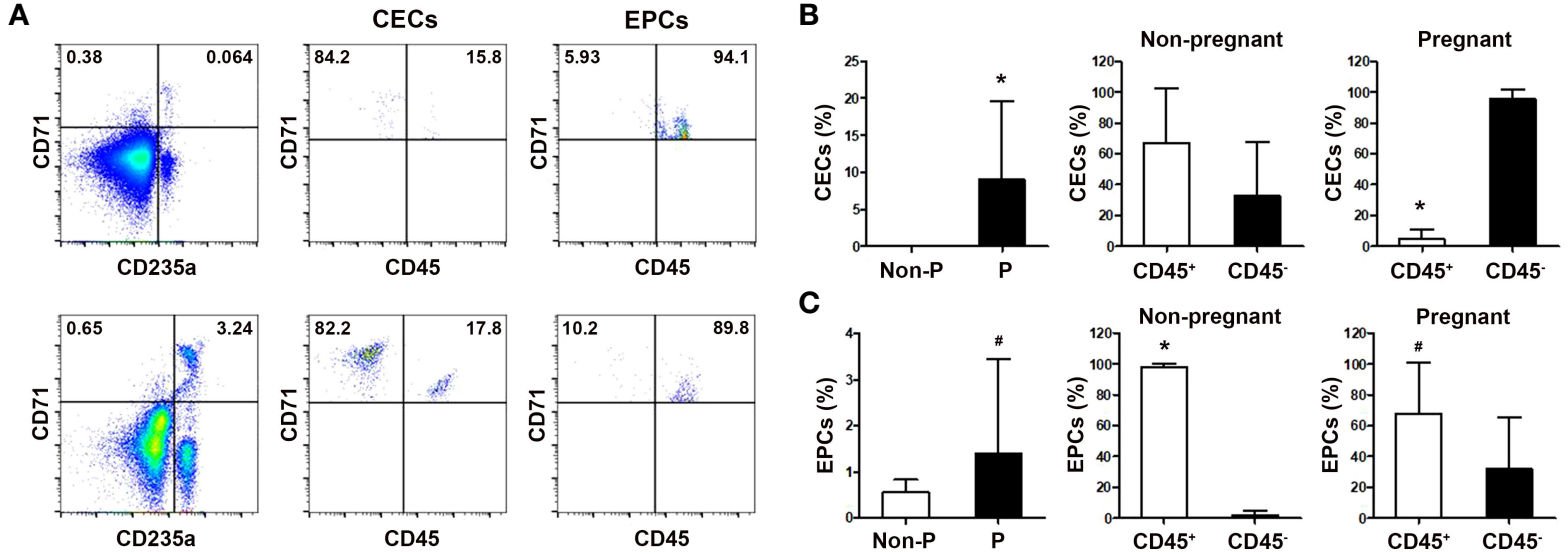
Comparison of the frequencies of circulating CECs in non-pregnant and pregnant women. **(A)** Representative flow cytometric scatter plots of circulating CD71^+^CD235a^+^ CECs, CD71^+^CD235a^-^ EPCs, and their respective CD45^+^ and CD45^-^ subgroups in non-pregnant (up) and pregnant women (down) were presented. Graphical summaries illustrating the percentages of CECs and their subgroups **(B)**, as well as EPCs and their subgroups **(C)**, in both non-pregnant and pregnant women were shown. Statistical analysis was performed using independent Student’s *t*-test and paired *t*-test. The results were expressed as mean ± SD. ^#^ *P* < 0.05 and ^*^ *P* < 0.01 vs. the control group. Non-P, non-pregnant women; P, pregnant women.

We next analyzed peripheral blood CECs frequencies in non-regnant and pregnant women. As shown in **Figure 2**, the frequencies of blood CD71^+^CD235a^+^ CECs and CD71^+^CD235a^-^ erythroid precursor cells (EPCs), which were more immature than CECs, were significantly increased in pregnant women compared with non-pregnant women (*P* < 0.01 for both comparisons). Interestingly, while the frequency of more immature CD45^+^ EPCs in the peripheral blood of pregnant women was significantly higher than their CD45^-^ counterpart, the frequency of more mature CD45^-^ CECs was significantly higher than that of CD45^+^ CECs during human pregnancy. These results suggest that CECs in pregnant women are more likely to be CD45^-^ mature cells.

### Comparison of the frequencies of HSPCs subsets and CECs in non-pregnant and pregnant mice

**Figure 3:**
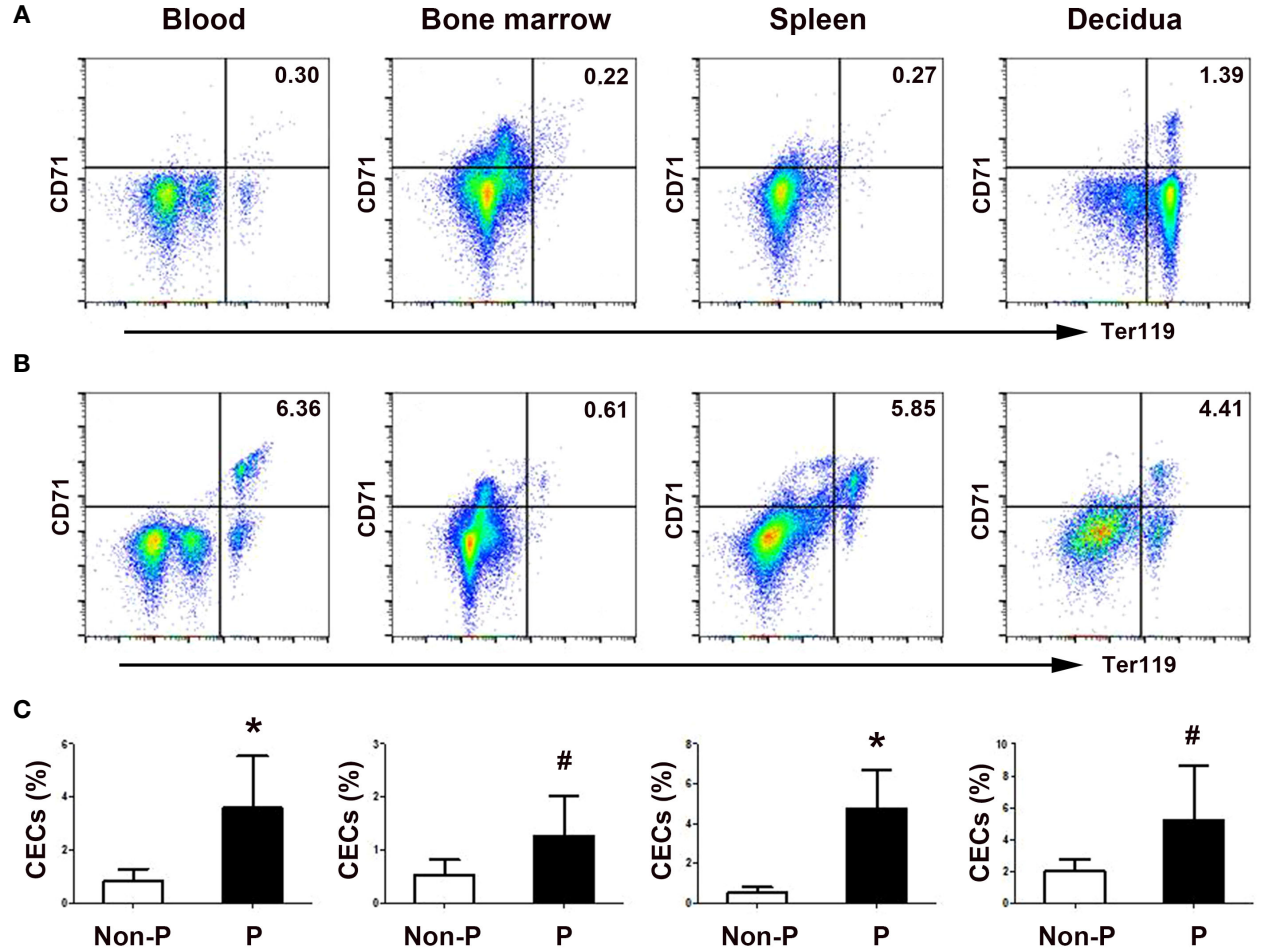
Physiological enrichment of CD71^+^Ter119^+^ CECs in pregnant mice. Representative flow cytometric scatter plots depicting CD71^+^Ter119^+^ CECs in the blood, bone marrow, spleen, and decidua between non-pregnant **(A)** and pregnant **(B)** mice were presented. **(C)** A graphical summary illustrating the percentages of CECs in non-pregnant and pregnant mice were shown. Statistical analysis was performed using independent Student’s *t*-test. The results were expressed as mean ± SD. ^#^ *P* < 0.05 and ^*^ *P* < 0.01 vs. the control group. Non-P, non-pregnant mice; P, pregnant mice.

In agreement with the results of human peripheral blood, the ratios of both LSK hemopoietic stem cells and Lineage^-^c-Kit^+^ (LK) hemopoietic progenitor cells to Lin^-^ cells, and the frequencies of MEPs and GMPs to LK cells significantly increased in the bone marrow of pregnant mice compared with non-pregnant female mice (*P* < 0.05, respectively; **Supplementary Figures 2A–C**). Similarly, the frequencies of splenic LSK cells, LK cells and MEPs in pregnant mice were also significantly increased compared with those in non-pregnant mice (*P* < 0.05, respectively; **Supplementary Figure 2D**). Besides, compared with non-pregnant female mice, pregnant mice had enlarged spleens (**Supplementary Figure 2E**). Meanwhile, we observed significant increases of CD71^+^TER119^+^ CECs in the blood (*P* < 0.01), bone marrow (*P* < 0.05), spleen (*P* < 0.01) and deciduae (*P* < 0.05) of pregnant mice compared with non-pregnant female mice (**Figure 3**). Our results indicate that hematopoiesis, especially erythropoiesis in both the bone marrow and spleen, is active in mouse pregnancy.

### EMH in the spleen served as a major source of increased CECs in pregnancy

**Figure 4:**
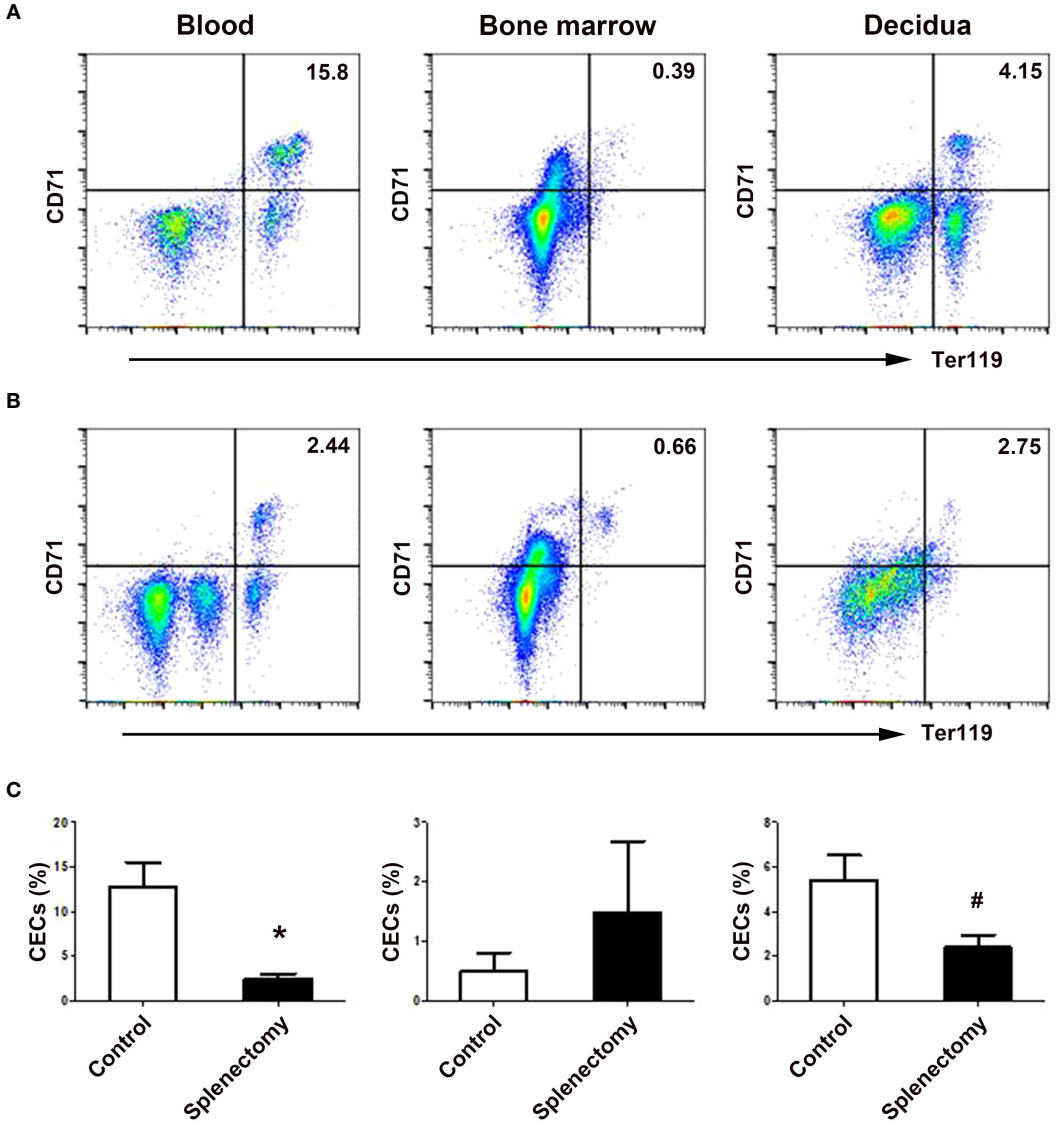
Splenectomy decreased the frequencies of CECs in the peripheral blood and deciduae of pregnant mice. Representative flow cytometric scatter plots of CD71^+^Ter119^+^ CECs in the blood, bone marrow and decidua between sham-operated **(A)** and splenectomized **(B)** pregnant mice were presented. **(C)** A graphical summary of the percentages of CECs in sham-operated and splenectomized pregnant mice were shown. Statistical analysis was performed using independent Student’s *t*-test. The results were expressed as mean ± SD. ^#^ *P* < 0.05 and ^*^ *P* < 0.01 vs. the sham-operated control group.

Since erythropoiesis was active in the mouse spleen, we investigated the role of EMH in the generation of CECs during mouse pregnancy. As shown in **Figure 4**, splenectomy significantly decreased the ratio of CD71^+^Ter119^+^ CECs in the peripheral blood (*P* < 0.01) and decidua (*P* < 0.05) of pregnant mice compared with the sham-operated group. However, splenectomy did not change the ratio of bone marrow CECs in pregnant mice. Our results demonstrate an important role of extramedullary erythropoiesis in the spleen but not in the bone marrow in the generation of increased CECs during mouse pregnancy.

### TGF-β promoted the generation of CECs from CD34^+^ HSPCs

To explore the mechanisms underlying the expansion of CECs during pregnancy, our investigation initially focused on the pregnancy-related hormones, which are crucial for the successful establishment and maintenance of pregnancy. Estradiol, progesterone and β-hCG showed no impact on the generation of CECs from human CD34^+^ HSPCs (**Supplementary Figure 3**).

**Figure 5:**
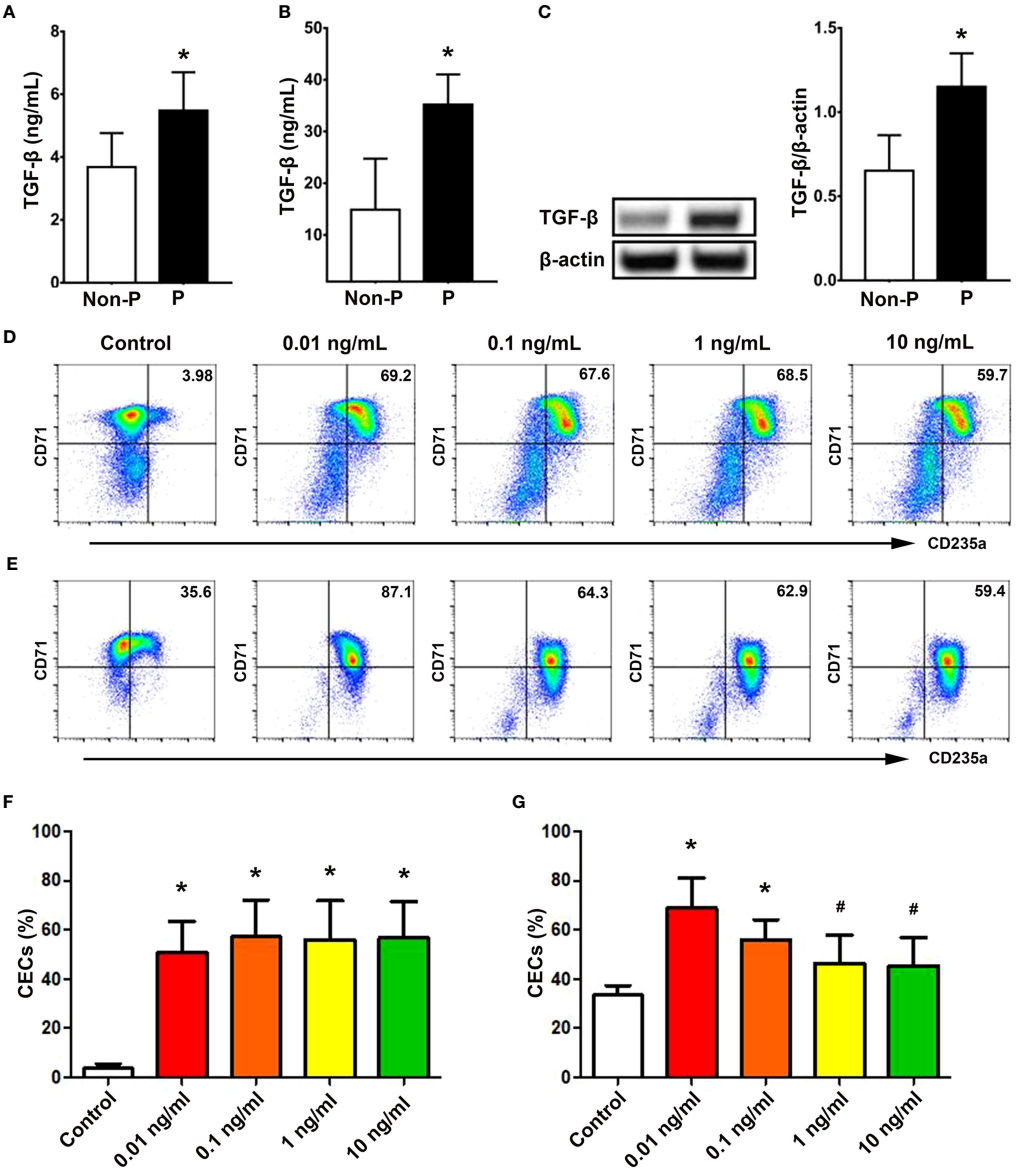
TGF-β promoted the generation of CECs from CD34^+^ HSPCs. Concentrations of TGF-β in the serum of non-pregnant and pregnant women **(A)** and mice **(B)** were presented. **(C)** Protein expression of splenic TGF-β in non-pregnant or pregnant mice was shown. Representative flow cytometric scatter plots of HSPCs-derived CECs treated with vehicle (present as *Control* group) or different concentrations of TGF-β on day 7 **(D)** and day 11 **(E)** were presented. Graphical summaries of the percentages of CECs from UBMC-derived HSPCs with vehicle or different concentrations of TGF-β on day 7 **(F)** and day 11 **(G)** were shown. Statistical analysis was performed using Student’s-*t* test or one-way ANOVA. The results were expressed as mean ± SD. ^#^ *P* < 0.05 and ^*^ *P* < 0.01 vs. the control group. Non-P, non-pregnant women or mice; P, pregnant women or mice.

Given the known significance of TGF-β signaling in erythropoiesis, which encompasses a dual effect on cellular proliferation and differentiation (28), we subsequently examined the involvement of TGF-β in erythropoiesis during pregnancy. As depicted in **Figure 5A**, the concentration of extracellular TGF-β in the serum of pregnant women was significantly higher compared to non-pregnant women (*P* < 0.01). Similarly, pregnant mice also exhibited elevated levels of serum TGF-β (*P* < 0.01; **Figure 5B**). Western blot analysis also demonstrated increased TGF-β in the spleen of pregnant mice compared with non-pregnant mice (*P* < 0.01; **Figure 5C**).

In addition, rhTGF-β at concentrations of 0.01, 0.1, 1, and 10 ng/mL significantly promoted the generation of CD71^+^CD235a^+^ CECs from UBMC-derived CD34^+^ HSPCs on day 7 (*P* < 0.01, respectively; **Figures 5D, F**) and on day 11 (*P* < 0.01, *P* < 0.01, *P* < 0.05 and *P* < 0.05; **Figures 5E, G**) compared with the control group without rhTGF-β. Meanwhile, as shown in **Supplementary Figure 4**, different concentrations (0.01, 0.1 and 1 ng/mL) of rhTGF-β significantly increased the mRNA levels of TGF-β downstream transcription factors, including *Smad2* (*P* < 0.01, respectively), *Smad3* (*P* < 0.05, respectively) and *Smad4* (*P* < 0.05, respectively), compared with the control group. At the same time, we investigated the effects of TGF-β on the proliferation of UBMC-derived CD34^+^ HSPCs. Different concentrations of TGF-β did not affect the percentages of EdU^+^ cells from UBMC-derived HSPCs in a proliferative culture system at 24 h (**Supplementary Figures 5A, C**) or 48 h (**Supplementary Figures 5B, D**).

### Splenic stromal cells promoted the production of CECs from LSK cells through the TGF-β pathway

**Figure 6:**
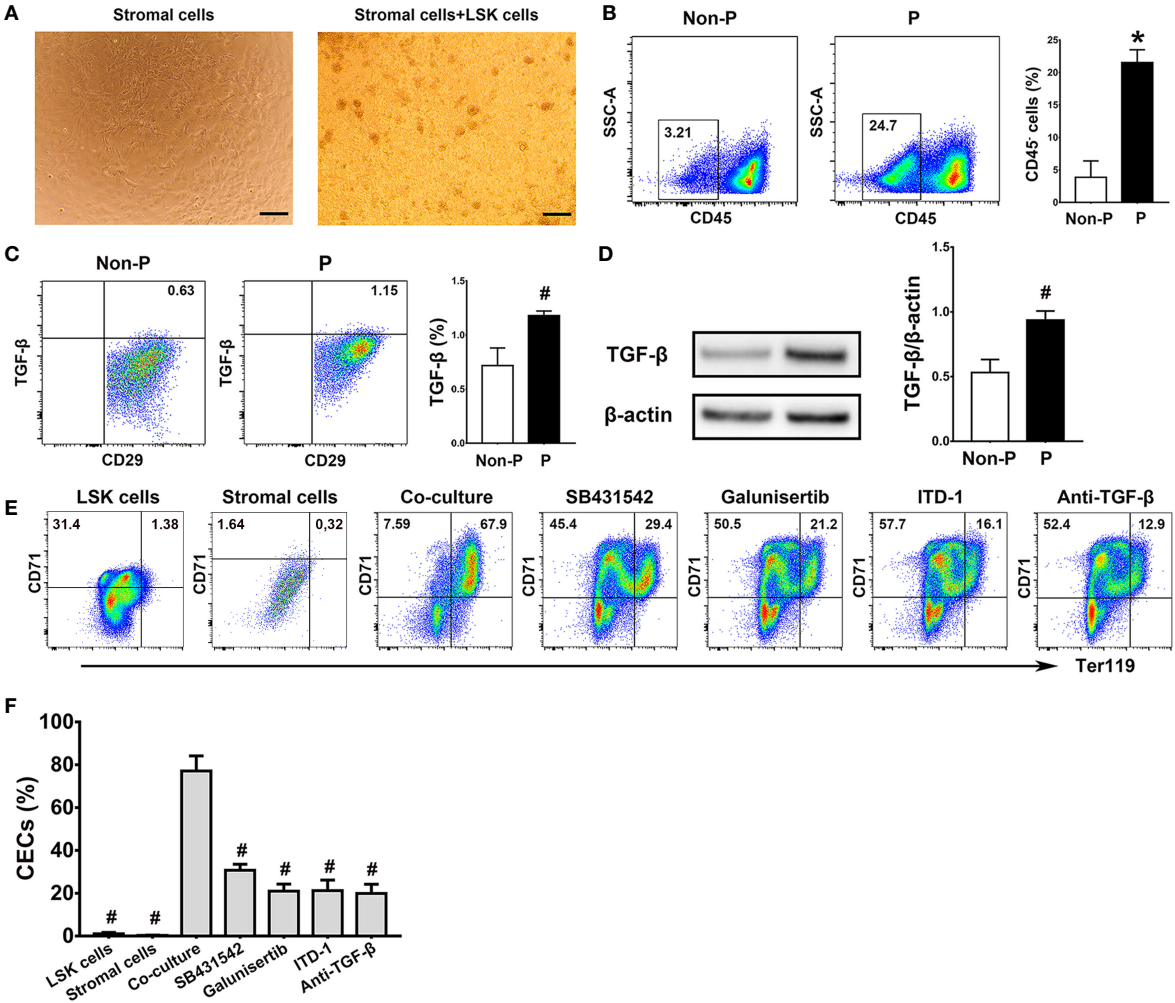
Splenic stromal cells promoted the production of CECs from LSK cells through the TGF-β pathway. **(A)** Representative morphologies of splenic stromal cells and the co-culture of bone marrow LSK cells and splenic stromal cells were shown. Scale Bar: 200 μm. Flow cytometric analysis of the percentages of CD45^-^ splenic stromal cells **(B)** and the production of intracellular TGF-β in splenic stromal cells **(C)** of non-pregnant or pregnant mice were presented. **(D)** The relative protein expression of splenic TGF-β in non-pregnant and pregnant mice assessed using Western blot analysis. **(E)** Flow cytometric scatter plots and graphical summaries were presented to illustrate the generation of CECs from co-cultures of LSK cells and splenic stromal cells, with or without different inhibitors of the TGF-β pathway. **(F)** The percentages of CECs in co-cultures of LSK cells and splenic stromal cells, with or without various inhibitors of TGF-β pathway, were displayed. Statistical analysis was performed using Student’s *t*-test or one-way ANOVA. The results were expressed as mean ± SD. ^#^ *P* < 0.05 and ^*^ *P* < 0.01 vs. the co-culture group. Non-P, non-pregnant mice; P, pregnant mice.

In consideration of elevated TGF-β in pregnancy and its significant effects on erythropoiesis, we further investigated the interactions between LSK cells and the splenic niche in TGF-β-induced erythropoiesis in the spleen. Co-culture of murine LSK cells with splenic stromal cells was established (**Figure 6A**). Cell clusters composed of differentiating erythroblasts were observed close to the adherent stromal cells in the co-cultures. In the meanwhile, we demonstrated significantly increased frequencies of splenic stromal cells (*P* < 0.01; **Figure 6B**) and upregulated intracellular TGF-β in splenic stromal cells using flow cytometry (*P* < 0.05; **Figure 6C**) and western blotting (*P* < 0.05; **Figure 6D**) in pregnant mice compared with non-pregnant mice. Compared with mono-culture of LSK cells or splenic stromal cells, co-culture of LSK cells and splenic stromal cells generated markedly increased percentages of CECs (*P* < 0.05 for both comparisons; **Figures 6E, F**). In addition, treating co-cultures with different inhibitors of TGF-β signaling, including TGF-β receptor inhibitors SB431542 and Galunisertib, a Smad2/3 inhibitor ITD-1 and an anti-mouse TGF-β Ab, significantly decreased the generation of CECs compared with the co-culture group alone (*P* < 0.05, respectively; **Figures 6E, F**). Our results suggest that splenic stromal cells significantly promoted the differentiation of LSK cells into CECs through the TGF-β pathway.

### Role of splenic macrophages in the generation of CECs from LSK cells

**Figure 7:**
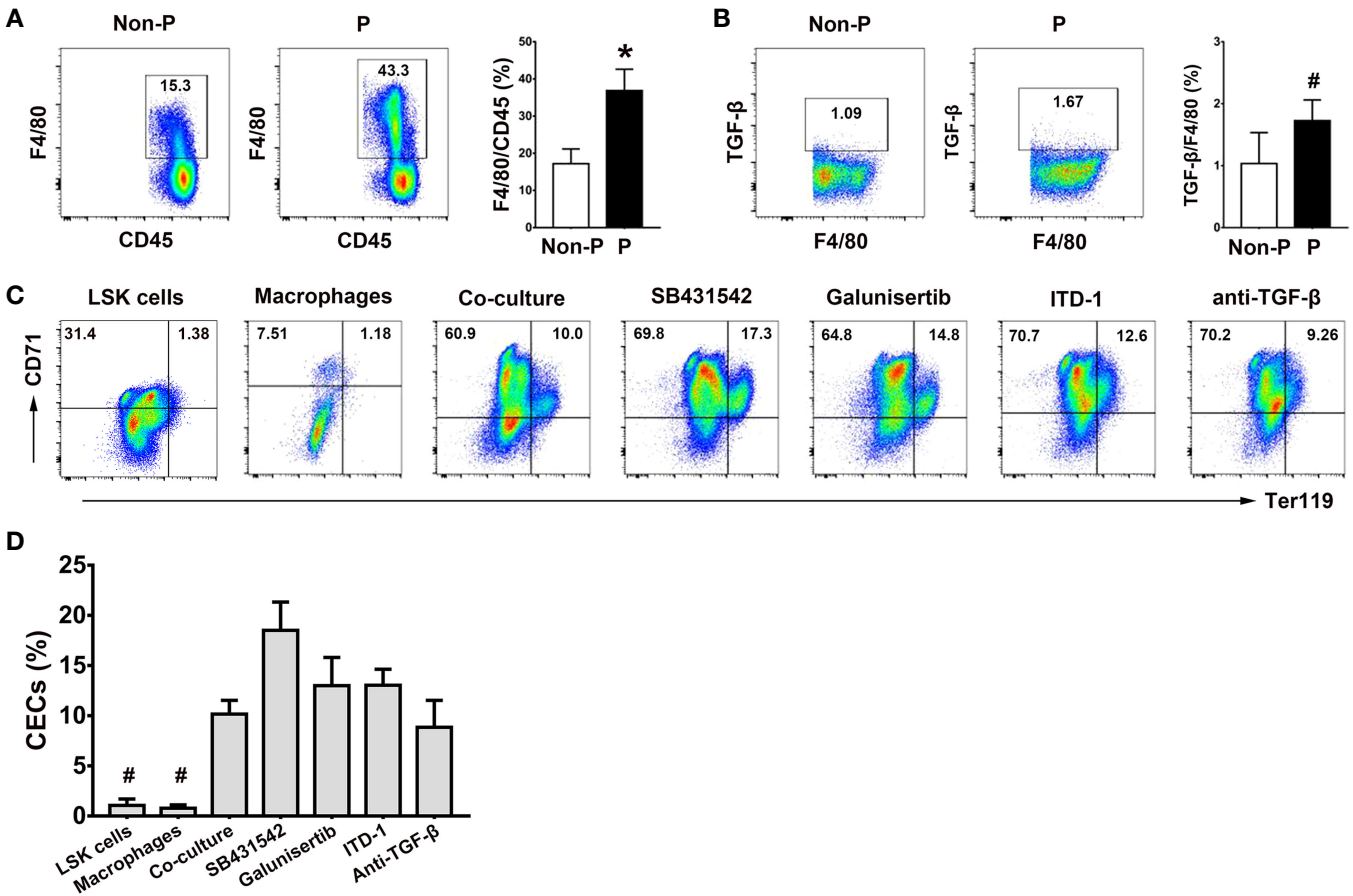
Role of splenic macrophages in the generation of CECs from LSK cells. **(A)** Representative flow cytometric scatter plots and a graphical summary of the percentages of splenic F4/80^+^ macrophages in non-pregnant and pregnant mice were shown. **(B)** Representative flow cytometric scatter plots and a graphical summary of the intracellular TGF-β production in splenic macrophages of non-pregnant or pregnant mice were presented. Representative flow cytometric scatter plots **(C)** and graphical summaries **(D)** of CECs generated from co-cultures of LSK cells and splenic macrophages with or without various TGF-β pathway inhibitors were shown. Statistical analysis was performed using Student’s *t*-test or one-way ANOVA. The results were expressed as mean ± SD. ^#^ *P* < 0.05 and ^*^ *P* < 0.01 vs. the co-culture group. Non-P, non-pregnant mice; P, pregnant mice.

Given the recognized significance of macrophages as a crucial element of the erythroblastic islands within the bone marrow and their integral involvement in erythropoiesis (29), our study sought to examine the contribution of splenic macrophages to extramedullary erythropoiesis by co-culturing LSK cells with splenic macrophages. Notably, we observed heightened proportions of splenic macrophages (*P* < 0.01; **Figure 7A**) and their intracellular TGF-β production (*P* < 0.05; **Figure 7B** *)* in pregnant mice in comparison to non-pregnant mice. Furthermore, the co-culture of LSK cells with splenic macrophages yielded a moderate enhancement in the generation of CECs from LSK cells, as opposed to the mono-culture of LSK cells or splenic macrophages alone (*P* < 0.05 for both comparisons; **Figures 7C, D**). However, blocking the TGF-β pathway with SB431542, Galunisertib, ITD-1 and an anti-mouse TGF-β Ab had no obvious impact on the generation of CD71^+^TER119^+^ cells (**Figures 7C, D**).

## Discussion

The process of erythropoiesis, which involves the generation of red blood cells, is highly efficient in the human body. It is estimated that approximately 2.5 million red blood cells are produced per second to maintain a balance with the removal of senescent erythrocytes (30, 31). However, under stress conditions, including blood loss (32), inflammation (33) and cancer (34), a regenerative process termed “stress erythropoiesis” is induced for rapid replenishment of erythrocytes (32). Erythroid progenitor cells and signals in stress erythropoiesis in both human and mice are different from those in steady-state erythropoiesis (35–37). To date, the specific properties of erythropoiesis during pregnancy have not been fully elucidated. Our study reveals that pregnant women exhibit elevated levels of HSPCs, EMPs, EPCs and CECs in comparison to non-pregnant women. Furthermore, pregnant mice demonstrate increased CECs in the blood, bone marrow, spleen, and decidua compared to non-pregnant mice, indicating active stress erythropoiesis during pregnancy. In addition, the upregulation of LSK cells, LK cells, MEPs, and CECs in the bone marrow and spleen of pregnant mice further supports the notion of enhanced erythropoiesis occurring in both of these organs.

During stress erythropoiesis, HSCs are mobilized from the bone marrow to peripheral sites in order to facilitate the expansion of hematopoiesis. Considering the scarcity of evidence substantiating extramedullary blood production, it is reasonable to postulate that these cellular reservoirs fulfill distinct roles during periods of hematopoietic demand. Studies primarily conducted in mice indicates the presence of various rare HSPCs populations outside the adult bone marrow (8). Although these populations do not appear to play a significant role in blood formation under normal conditions, they likely serve as emergency reservoirs that promptly respond to stress. Nevertheless, our comprehension of extramedullary hematopoiesis and HSPCs, particularly in humans, remains relatively restricted. Further investigation focused on understanding human stress erythropoiesis will yield substantial benefits for patients and offer novel approaches to enhance or regulate erythropoiesis. Stress erythropoiesis is most comprehensively understood in mice and predominantly occurs in the spleen (11). Our study observed a significant decrease in the percentage of CECs from peripheral blood and decidua, but not bone marrow, in pregnant mice following splenectomy, along with the presence of enlarged spleens in pregnant mice. These findings suggest a potential involvement of stress erythropoiesis in pregnancy, which mainly occurs in the spleen.

HSPCs and their surrounding tissue microenvironment are essential constituents of the hematopoietic system. This specialized microenvironment, referred to as the niche, plays a pivotal role in facilitating the processes of self-renewal, differentiation, proliferation, and migration of HSPCs under both normal hematopoietic homeostasis and pathological stress conditions (7). In the bone marrow niche, the erythroblastic island, consisting of a central resident macrophage surrounded by differentiating erythroblasts (30, 38), is responsible for maintaining the continuous production of red blood cells. This microenvironment provides essential nutritional and survival support to ensure efficient erythropoiesis (39). Additionally, extramedullary erythropoiesis predominantly takes place in the red pulp of the spleen, where stress erythropoietic progenitors are primarily sustained by endothelial cells located around sinusoids and stromal cells (9). It has been demonstrated that CXCL12 and SCF produced by splenic stromal cells surrounding sinusoids in the red pulp are critical for the recruitment and maintenance of HSCs during EMH (40). However, the molecular mechanisms of extramedullary erythropoiesis in pregnancy have thus far remained unclear. Co-culture of mouse bone marrow LSK cells with splenic stromal cells significantly increased the differentiation of LSK cells toward CECs in our study, suggesting that splenic stromal cells are an essential niche for extramedullary erythropoiesis in pregnancy.

The role of TGF-β signaling in erythropoiesis is crucial yet subject to debate. TGF-β is responsible for halting the proliferation of BFU-Es, while promoting the differentiation of early BFU-Es into late BFU-Es and CFU-Es, and CFU-Es into more mature stages, in collaboration with EPO (41). Furthermore, studies have demonstrated that TGF-β inhibitors enhance the self-renewal of BFU-E progenitors, leading to an increase in red blood cell production (26). However, the functions of TGF-β are more complex and largely context-dependent. It has been demonstrated that TGF-β at low concentrations leads to the stimulation of colony formation from CD34^+^ cells (42). Moreover, the use of neutralizing Abs against TGF-β has been found to inhibit subsequent erythropoiesis in an erythroid cell culture, suggesting the presence of a stimulatory effect exerted by autocrine TGF-β (43). In addition, HSC lacking the TGF-β type II receptor demonstrate reduced Smad activation and impaired long-term repopulating activity, highlighting the crucial role played by the TGF-β/Smad pathway in HSC maintenance (44). Besides, a low concentration of TGF-β does not hinder the proliferation of the early erythropoiesis stages. Conversely, it significantly expedites the terminal stages of erythroid differentiation through the facilitation of BNIP3L/NIX-mediated mitophagy (45). TGF-β is consistently synthesized by bone marrow stromal cells and has the ability to directly stimulate the production of regulatory T cells (Tregs) in a manner dependent on monocytes (46, 47). When human bone marrow stromal cells are co-cultured with PBMCs *in vitro*, it leads to the development of CD4^+^ T cells into induced-Tregs expressing CD25^+^FoxP3^+^. This process involves direct interaction between stromal cells and helper T cells, as well as the secretion of PGE2 and TGF-β (48). In our study, the elevation of TGF-β in serum and spleen, along with the increased expression of TGF-β in splenic stromal cells, and decreased generation of CECs from co-cultures of LSK cells and splenic stromal cells upon treatment with various inhibitors of TGF-β signaling, strongly suggests that TGF-β plays a crucial role in the production of CECs from LSK cells induced by splenic stromal cells. Meanwhile, it was observed that TGF-β did not exhibit any inhibitory impact on the proliferation of CD34^+^ HSPCs under proliferative culture conditions.

Macrophages are extensively distributed within splenic perivascular niches and hold significant significance in splenic erythropoiesis during specific stress circumstances (49–52). We also found their role in promoting CECs differentiation from LSK cells. However, their effects were weaker than those of splenic stromal cells, at least *in vitro*. Their effects on promoting extramedullary erythropoiesis *in vivo* are worthy of further study. Although the frequencies of splenic macrophages and their intracellular TGF-β significantly increased during pregnancy, their contribution to erythropoiesis is TGF-β-independent since treatment with diverse inhibitors of TGF-β signaling did not affect the generation of CECs from co-cultures of LSK cells and splenic macrophages compared with co-cultures without inhibitors. Some transcription factors or signaling pathways other than TGF-β signaling may play a role in extramedullary erythropoiesis by splenic macrophages, and need further investigation.

In conclusion, we found enhanced erythropoiesis and increased CECs in both human and mouse pregnancies. Increased TGF-β in pregnancy promoted the differentiation of CD34^+^ HSPCs to CECs, but did not affect the proliferation of HSPCs, resulting in eventual enhanced erythropoiesis. Elevated CECs in pregnancy were mainly derived from EMH in the spleen. Splenic stromal cells significantly enhanced splenic erythropoiesis through TGF-β signaling, while splenic macrophages contributed to extramedullary erythropoiesis in a TGF-β-independent manner during mouse pregnancy.

## Data availability statement

The original contributions presented in the study are included in the article/**Supplementary Material**. Further inquiries can be directed to the corresponding author.

## Ethics statement

The studies involving humans were approved by Institutional Review Board of Shenzhen People’s Hospital. The studies were conducted in accordance with the local legislation and institutional requirements. The participants provided their written informed consent to participate in this study. The animal study was approved by the Animal Care and Use Committee of Jinan University. The study was conducted in accordance with the local legislation and institutional requirements.

## Author contributions

YF: Conceptualization, Data curation, Formal Analysis, Investigation, Methodology, Software, Writing – original draft, Writing – review & editing. ZL: Investigation, Methodology, Writing – original draft, Writing – review & editing. WL: Investigation, Methodology, Writing – original draft, Writing – review & editing. XJ: Writing – review & editing. QS: Writing – review & editing. XM: Writing – review & editing. JY: Investigation, Methodology, Writing – review & editing. JT: Writing – review & editing. XL: Writing – review & editing. LL: Conceptualization, Funding acquisition, Methodology, Project administration, Supervision, Writing – original draft, Writing – review & editing.

## Glossary

**Table:**

CECs	CD71+ erythroid cells
EPCs	Erythroid precursor cells
EMH	Extramedullary hematopoiesis
HSCs	Hematopoietic stem cells
HSPCs	Hematopoietic stem and progenitor cells
MEPs	Megakaryocyte-erythrocyte progenitors
CMPs	Common myeloid progenitors
MPPs	Multipotent progenitors
GMPs	Granulocyte-monocyte progenitors
CLPs	Common lymphoid progenitors
BFU-E	Burst-forming unit-erythroid progenitors
CFU-E	Colony-forming unit erythroid progenitors
TGF-β	Transforming growth factor β
β-hCG	Human chorionic gonadotropin β
EPO	Erythropoietin
SCF	Stem cell factor
IL-3	Interleukin-3
CD71	Transferrin receptor I
CD235a	Glycoprotein A
Ter119	Glycoprotein A-related protein
PBMCs	Peripheral blood mononuclear cells
UBMCs	Umbilical cord blood mononuclear cells
RT-PCR	Real time polymerase chain reaction
LSK	Lineage^-^Sca-1^+^c-Kit^+^
LK	Lineage^-^c-Kit^+^
FBS	Fetal bovine serum
Flt-3	Fms-like tyrosine kinase 3
TPO	Thrombopoietin
ELISA	Enzyme-linked immunosorbent assay
