# Supplementary material for: Extramedullary hematopoiesis contributes to enhanced erythropoiesis during pregnancy via TGF-β signaling
Source: Front Immunol. 2023 Nov 17;14:1295717. doi: 10.3389/fimmu.2023.1295717 (PMC10693449; doi:10.3389/fimmu.2023.1295717)
Supplement: Supplementary file 1 [file DataSheet_1.pdf]

Supplementary Information
Supplementary Materials and Methods

Isolation of mouse PBMCs
Peripheral blood samples were collected from the vena orbitalis, heparinized, and purified by centrifugation over the Ficoll-Paque Premium (1.077 g/mL density gradient medium; Cytiva, Piscataway, NJ, USA) at 400 g for 30 min at 20°C. PBMCs were collected and washed. The cells were filtered through a 40 μm cell strainer (Thermo Fisher Scientific, Waltham, MA, USA) and re-suspended for further analysis.

Isolation of mouse spleen mononuclear cells and macrophages
Mouse spleen was collected, cut into small pieces and mashed through a 40 μm cell strainer to obtain single-cell suspensions. Mononuclear cells were then enriched by lysing red cells using a red cell lysis buffer (Biolegend, San Diego, CA, USA). In some experiments, spleen macrophages were isolated from the spleen mononuclear cells. In brief, cells were stained with fluorescence-conjugated Abs against CD45, CD3 and F4/80 (BioLegend), and $CD45^{+}CD3^{-}$ $F4/80^{+}$ cells were sorted using the Sony LE-SH800ZBP flow cytometer (Tokyo, Japan). Sorted cells were analyzed using flow cytometry, and the cell purity was > 99%.

Isolation of mouse bone marrow mononuclear cells
Mouse bone marrow cells were extracted from the marrow cavities of mouse femurs and humeri by repeated flushing PBS into the marrow cavities. Mononuclear cells were then collected and enriched by lysing red cells using a red cell lysis buffer (BioLegend). The cells were filtered through a 40 μm cell strainer and re-suspended for further analysis.

Isolation of mouse decidua mononuclear cells
Mouse uterine tissues were collected, washed, cut into small pieces, and digested using 1 mg/mL collagenase IV (Sigma-Aldrich, St. Louis, MO, USA) and 0.1 mg/mL DNase I (Roche, Basel, Switzerland) at 37°C for about 20 min in a shaking water bath. When single or clumps of cells were observed under the microscope, released cells were separated from undigested tissue pieces by filtering through a 40 μm cell strainer. Mononuclear cells were purified over the Ficoll-Paque Premium by centrifugation at 400 g for 30 min at 20°C. Mononuclear cells were collected, washed, and re-suspended for further analysis.

Quantitative RT-PCR
Total tissue RNA was extracted using the NucleoSpin RNA Plus kit (Macherey-Nagel, Düren, Germany) according to the manufacturer's protocol. The yield and purity of total RNA were determined using the Nanodrop2000 kit (Thermo Fisher Scientific). Subsequently, 1 μg of total RNA was reverse-transcribed into cDNA using the iScript cDNA Synthesis kit (Bio-Rad, Hercules, CA, USA) according to the manufacturer's instructions. RT-PCR was performed using the iQ SYBR Green Supermix Kit (Bio-Rad) with the StepOnePlus Real-time PCR System (Applied Biosystems, Waltham, MA). The primers were listed in Additional file 1: Table S2. The mRNA levels were measured with GAPDH (Sangon, Shanghai, China) as the internal reference.

Proliferation assay
The proliferation of UBMC-derived $CD34^{+}$ HSPCs was analyzed using a Flour488 Click-iT EdU Flow Cytometry Assay kit according to the manufacturer's instructions (KeyGEN Biotech, Nanjing, Jiangsu, China). In brief, UBMC-derived $CD34^{+}$ cells were cultured at a concentration of 1×10^5 cells/mL in the 12-well plate with proliferation medium containing the StemSpan SFEM II (Stem Cell Technologies, Vancouver, Canada) supplemented with 100 ng/mL SCF (PeproTech, Rocky Hill, NJ, USA), 100 ng/mL Flt-3 (BioLegend), and 100 ng/mL

(PeproTech) with or without different concentrations of rhTGF-β (R&D Systems, Minneapolis, MN, USA) for three days. EdU was added into the culture at the final concentration of 5 μM, and cells were cultured for another 24 or 48 h. Flow cytometry was performed to measure the cell proliferation based on Alex Flour 488 (EdU) positive cells. Each set of experiments was replicated three times.

Supplementary Tables

Table S1. Technical specifications of Abs used in this study

Antigen	Specificity	Conjugation	Clone	Source	Cat. No.
Lineage cocktail	Human	FITC	Mix	BioLegend	348801
CD34	Human	APC	561	BioLegend	343608
CD38	Human	PE	HIT2	BioLegend	303506
CD123	Human	PE/Cy7	6H6	BioLegend	306010
CD45RA	Human	APC/Cy7	HI100	BioLegend	304128
CD90	Human	PerCP/Cy5.5	5E10	BioLegend	328118
CD127	Human	BV605	A019D5	BioLegend	351334
CD45	Human	FITC	HI30	BioLegend	304006
CD45	Human	APC/Cy7	HI30	BioLegend	304014
CD71	Human	APC	CY1G4	BioLegend	334108
CD235a	Human	PE	HI264	BioLegend	349106
Lineage cocktail	Mouse	FITC	Mix	BioLegend	133301
CD117	Mouse	APC-Cy7	2B8	BioLegend	105826
Sca-1	Mouse	BV421	D7	BioLegend	108127
Sca-1	Mouse	PE	D7	BioLegend	108108
CD16/CD32	Mouse	AF647	93	BioLegend	101314
CD34	Mouse	PE	MEC14.7	BioLegend	119308
CD45	Mouse	APC/Cy7	30-F11	BioLegend	103116
CD45	Mouse	FITC	30-F11	BioLegend	103108

CD71	Mouse	APC	RI7217	BioLegend	113820
TER119	Mouse	FITC	TER-119	BioLegend	116206
CD3	Mouse	BV421	145-2C11	BioLegend	100336
F4/80	Mouse	AF700	BM8	BioLegend	123130
TGF-β1	Mouse	APC	TW7-16B4	BioLegend	141406
CD29	Mouse	AF647	HMβ1-1	BioLegend	102214

Table S2. Primers used for quantitative RT-PCR

Gene Name	Primer sequence	Product size (bp)
GAPDH	F: 5'-TGACTTCAACAGCGACAC-3'	199
	R: 5'-TCTTCCTCTTGTGCTCTTG-3'	
Smad2	F: 5'-ATCACGAGGTCAGGAGTT-3'	148
	R: 5'-TCAGGTTCAAGCCAATCTT-3'	
Smad3	F: 5'-GCTGCTTGTAGGACTGTT-3'	162
	R: 5'-CTTCGCTGTGTTGAGGTT-3'	
Smad4	F: 5'-TTCCTGATTCTTGCTGAGTT-3'	168
	R: 5'-CTCTGGCTACTGATGCTAAT-3'	

3

Supplementary Figures

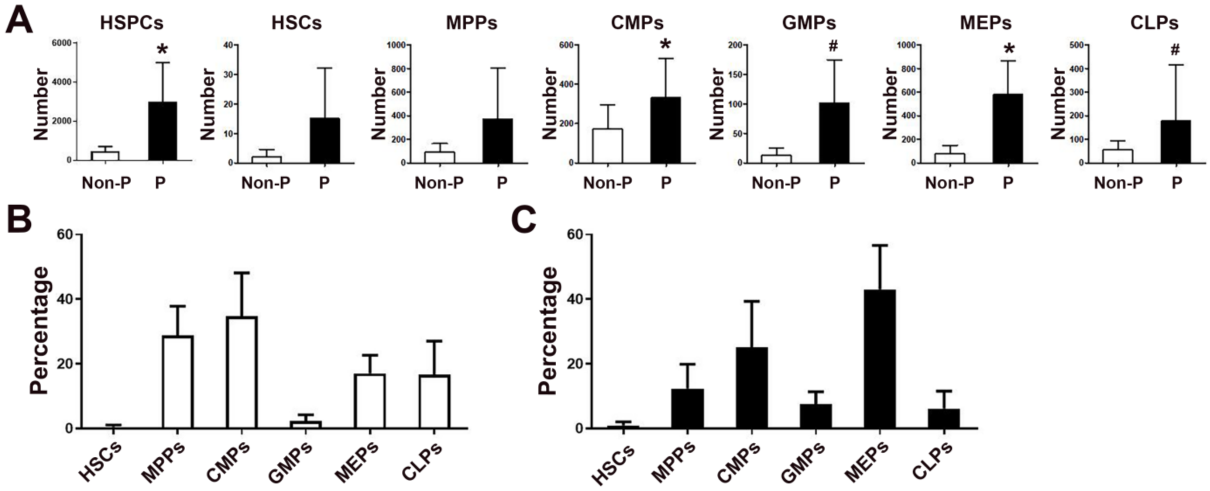

Figure S1. Comparison of the numbers of circulating HPSCs and their subgroups, and their components in the peripheral blood of non-pregnant and pregnant women. (A) A graphical summary of the numbers of HSPCs and their subgroups in the peripheral blood of non-pregnant and pregnant women were presented. Graphical summaries of the percentages of HSPC components in the peripheral blood of non-pregnant women **(B)** and pregnant women **(C)** were shown. Data were analyzed using independent Student's *t*-test. Results were expressed as mean ± SD. $^{\#}P < 0.05$ and $^{*}P < 0.01$ vs the control group. Non-P: non-pregnant women; P: pregnant women.

4

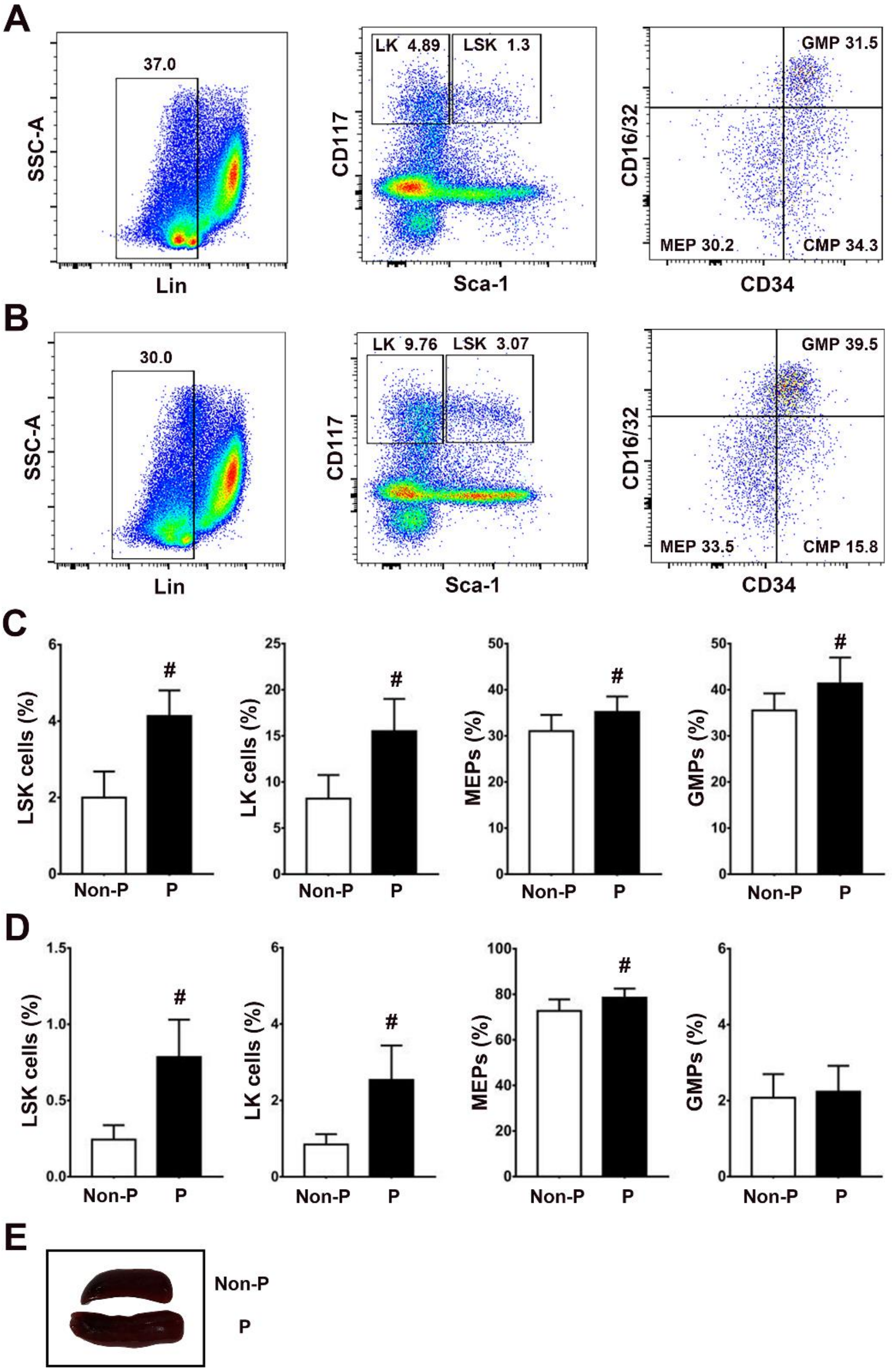

Figure S2. Altered composition of HSPC subsets in pregnant mice. Representative flow cytometric scatter plots of HSPCs and their subgroups in the bone marrow of non-pregnant **(A)** and pregnant mice **(B)** were presented. Graphical summaries of the percentages of HSPCs and their subgroups in mouse bone marrow **(C)** and spleen **(D)** were shown. **(E)** The spleens of non-pregnant and pregnant mice were shown. Data were analyzed using independent Student's *t*-test. Results were expressed as mean ± SD. $^{\#}P < 0.05$ vs. the control group. Non-P: non-pregnant mice; P: pregnant mice.

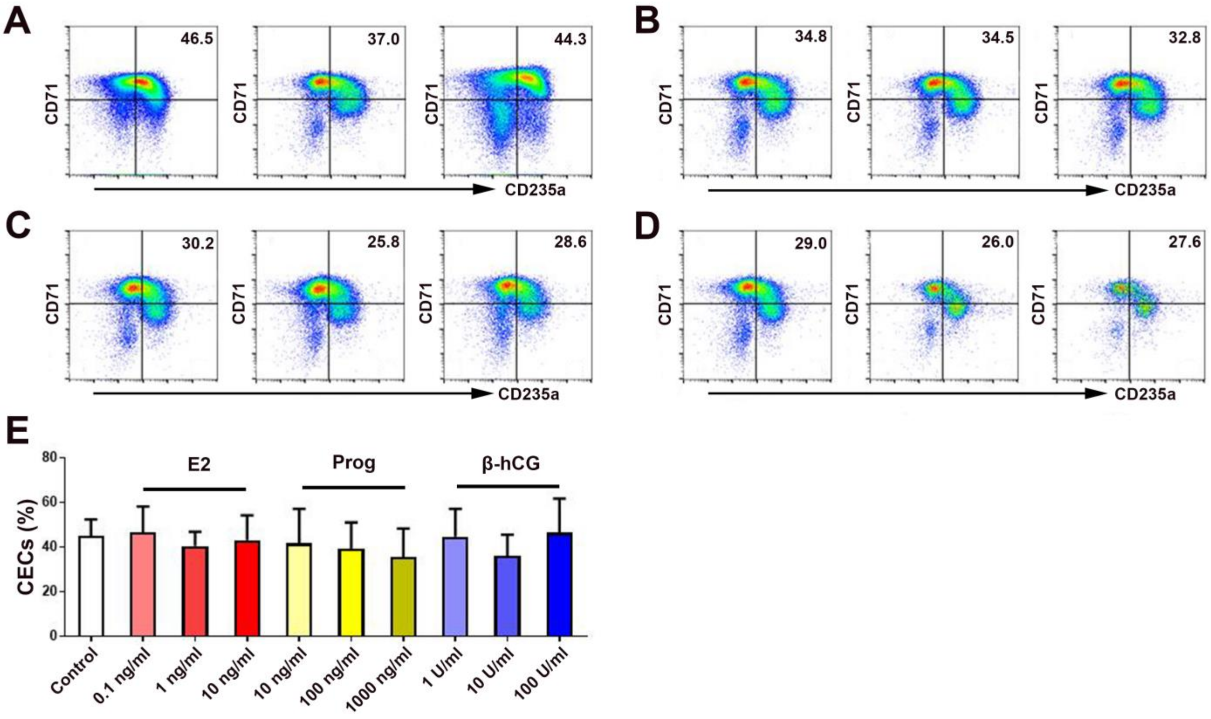

Figure S3. Effects of pregnancy-related hormones on the generation of CECs from CD34$^+$ HSPCs. Representative flow cytometry scatter plots of HSPCs-derived CECs treated with vehicle (present as *Control* group) **(A)** or different concentrations of estradiol **(B)**, progesterone **(C)**, and β-hCG **(D)** were shown. Graphical summaries of the percentages of CECs from UBMC-derived HSPCs with vehicle or different concentrations of pregnancy-related hormones **(E)** were presented. Data were analyzed using one-way ANOVA. Results were expressed as mean ± SD. E2: estradiol. Prog: progesterone.

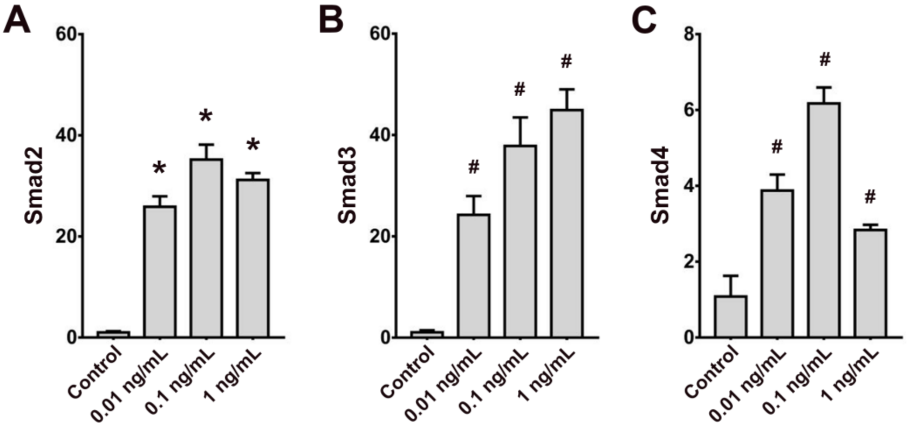

Figure S4. TGF-β downstream pathways during TGF-β-induced erythropoiesis. Relative mRNA levels of *Smad2* **(A)**, *Smad3* **(B)**, and *Smad4* **(C)** expressed by TGF-β-treated CD34$^+$ HSPCs cultured under erythroid differentiation conditions. Cells treated with vehicle present as *Control* group. Data were analyzed using one-way ANOVA. Results were expressed as mean ± SD. $^{\#}P < 0.05$ and $^{*}P < 0.01$ vs. the control group.

6

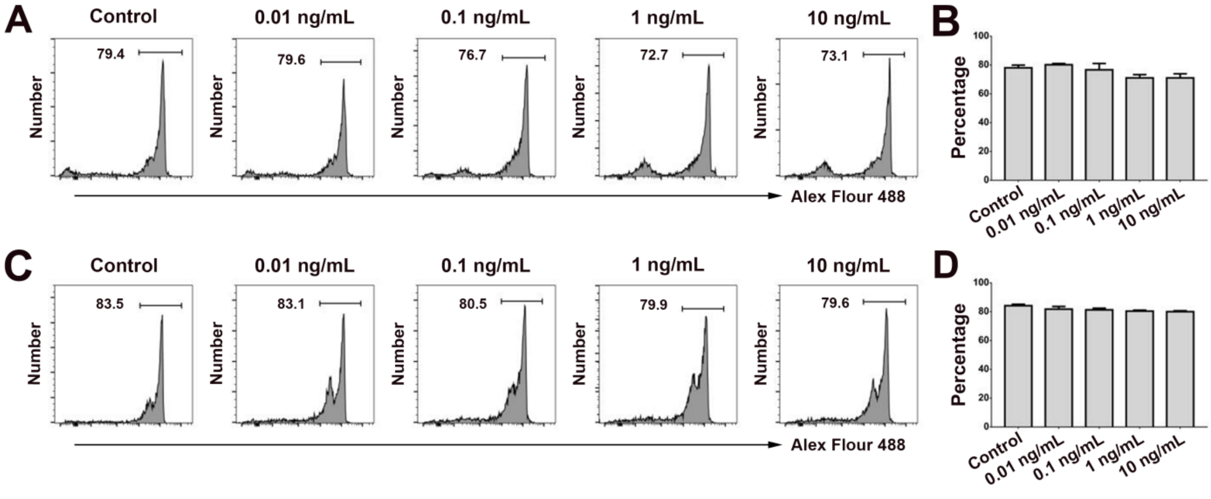

Figure S5. Effects of TGF-β on the proliferation of UBMC-derived CD34$^+$ HSPCs. Representative flow cytometric histograms of Alex Flour 488$^+$ cells from UBMC-derived CD34$^+$ HSPCs in proliferation media supplemented with different concentrations of TGF-β at 24 h **(A)** or 48 h **(C)** were shown. Cells treated with vehicle present as *Control* group. Graphical summaries of the percentages of Alex Flour 488$^+$ cells from UBMC-derived HSPCs in proliferation media supplemented with different concentrations of TGF-β at 24 h **(B)** or 48 h **(D)** were presented. Data were analyzed using one-way ANOVA. Results were expressed as mean ± SD.

7
